# Takotsubo syndrome associated with autoimmune limbic encephalitis: a case report

**DOI:** 10.1186/s12872-020-01789-3

**Published:** 2021-02-11

**Authors:** Yuki Kakinuma, Taro Kimura, Yoshiki Sakae, Satomi Kubota, Kenjiro Ono, Ryuta Kinno

**Affiliations:** 1 Division of Neurology, Department of Internal Medicine, Showa University Northern Yokohama Hospital, 35-1 Chigasaki-chuo, Tsuzuki-ku, Yokohama-shi, Kanagawa, 224-8503 Japan; 2 Division of Cardiology & Cardiac Catheterization Laboratories, Showa University Northern Yokohama Hospital, 35-1 Chigasaki-chuo, Tsuzuki-ku, Yokohama-shi, Kanagawa, 224-8503 Japan; 3 Division of Neurology, Department of Medicine, Showa University, School of Medicine, 1-5-8 Hatanodai, Shinagawa-ku, Tokyo, 142-8666 Japan

**Keywords:** Autoimmune limbic encephalitis, Case report, Limbic system, Stress cardiomyopathy, Takotsubo syndrome

## Abstract

**Background:**

Central nervous system diseases are common triggers of Takotsubo syndrome. We herein report a rare case of Takotsubo syndrome associated with autoimmune limbic encephalitis.

**Case presentation:**

A 68-year-old Japanese woman presented to our emergency room with disturbed consciousness. At admission, she showed hypoxemia. Left ventriculography showed akinesia in the middle part of the left ventricle and hyperkinesia in the apical and basal parts of the left ventricle, and the diagnosis of midventricular Takotsubo syndrome was established. However, after an improvement in disturbed consciousness and Takotsubo syndrome symptoms, her brother noticed something wrong with her behavior during his visit to the hospital. Subsequently, we consulted the neurology department 1 week after admission. Her brother revealed a history of abnormal behavior by the patient (such as mistaken entry in the wrong apartment in her building or in another person’s car) a few days prior to the onset of disturbed consciousness, suggesting disorientation of place. Brain magnetic resonance imaging showed an increased signal in the medial aspect of the temporal lobes, which was most clearly observed on the fluid-attenuated inversion recovery sequence; additionally, a cerebrospinal fluid analysis revealed mild lymphocytic pleocytosis. Finally, we established a diagnosis of midventricular Takotsubo syndrome associated with autoimmune limbic encephalitis.

**Conclusions:**

It is presumed that the dysfunction of limbic system due to autonomic limbic encephalopathy is associated with exaggerated sympathetic stimulation. This likely resulted in Takotsubo syndrome in our patient.

## Background

Takotsubo syndrome is characterized by transient systolic and diastolic left ventricular dysfunction with a variety of wall-motion abnormalities [1]. The condition predominantly affects elderly women and is often triggered by emotional or physical stimuli [2]. Central nervous system diseases are common triggers of Takotsubo syndrome [3]. Here we report a rare case of midventricular Takotsubo syndrome associated with autoimmune limbic encephalitis. Dysfunction of the limbic system due to autoimmune limbic encephalitis may be associated with Takotsubo syndrome.

## Case presentation

**Fig. 1:**
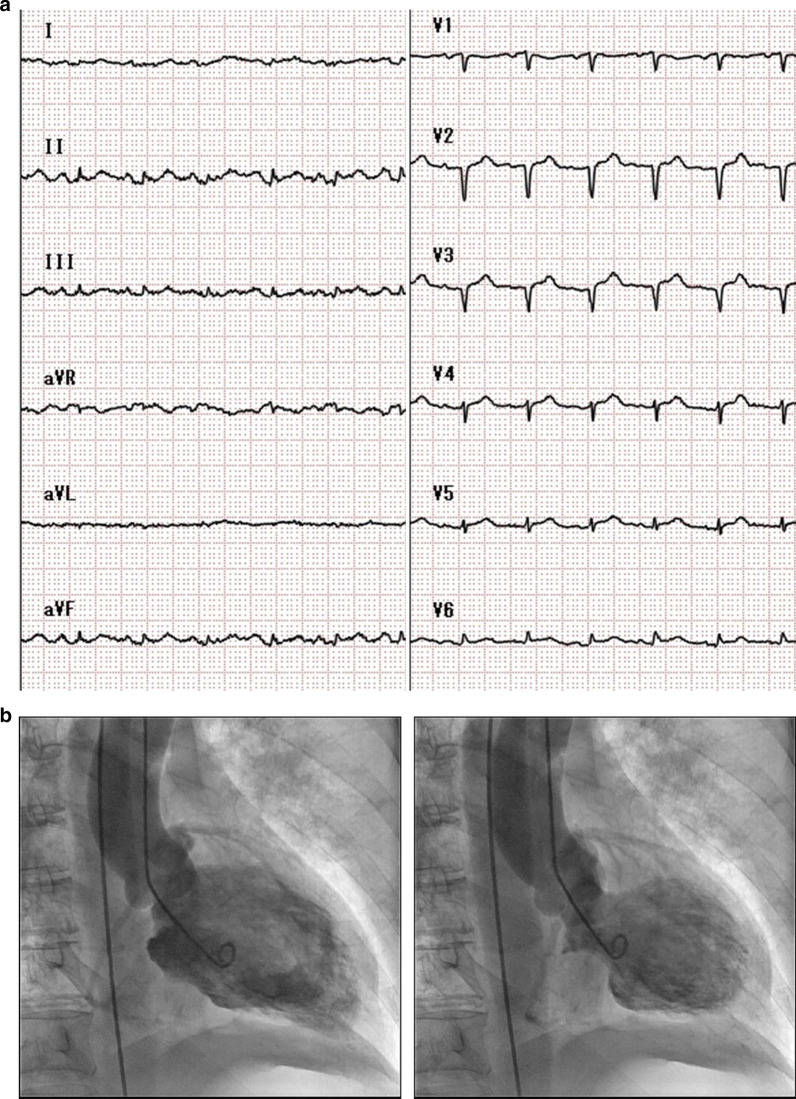
Cardiac findings. **a**: ECG findings. Note that there is no ST-segment depression. **b**: Left ventriculography findings. Akinesia in the middle section of the left ventricle and hyperkinesia in the apical and basal sections of the left ventricle during diastole (left) and systole (right) are observed

A 68-year-old Japanese woman presented to our emergency room with disturbed consciousness. She had a history of subclinical hypothyroidism and was not receiving any medication. Her family history was unremarkable. At admission, her temperature, blood pressure, and pulse rate were 37.8 °C, 126/88 mmHg, and 123 beat per minutes, respectively. She showed low levels of arterial oxygen saturation (84%), and a chest x-ray showed diffuse pulmonary edema. A blood gas analysis showed hypoxia (PO_2_: 56.2 mmHg). Her electrocardiogram (ECG) showed a sinus rhythm with subtle and nonspecific ST-segment elevation in all leads (Fig. 1a). Importantly, neither ST-segment depression nor QTc prolongation (340 ms) was observed in her ECG. At this time, her InterTAK Diagnostic Score [4] was 37 (Female Sex [25 points], No ST-segment depression [12 points]). The blood tests showed mildly elevated levels of creatine kinase-MB isoenzyme (85 U/L; normal: < 25 U/L), and troponin T (0.030 ng/mL; normal: < 0.014 ng/mL), as well as elevated level of brain natriuretic peptide (BNP: 764.9 pg/mL; normal: < 18.4 pg/mL), suggesting a possible diagnosis of acute coronary syndrome and acute heart failure. Transthoracic echocardiography showed left ventricular systolic dysfunction (ejection fraction [EF], 28%), akinesis in the middle section of the left ventricle, and preserved contractility of the apical and basal sections of the left ventricle. On emergent cardiac catheterization, coronary arteriography showed no significant stenosis, and the ergonovine provocation test yielded negative results. Left ventriculography showed akinesia in the middle part of the left ventricle and hyperkinesia in the apical and basal parts of the left ventricle, resulting in a midventricular Takotsubo syndrome (Fig. 1b, See Additional file 1). We initiated respiratory care with noninvasive positive pressure ventilation (NPPV), intravenous furosemide, and carperitide for the treatment of acute heart failure with pulmonary edema. Four days after the onset, her disturbed consciousness and cardiac symptoms rapidly improved, and NPPV was discontinued. Accordingly, we started an angiotensin-converting enzyme inhibitor (imidapril 5 mg/day). She showed almost normal levels of BNP (19.5 pg/ml). The clinical course was consistent with the diagnosis of midventricular Takotsubo syndrome.

**Fig. 2:**
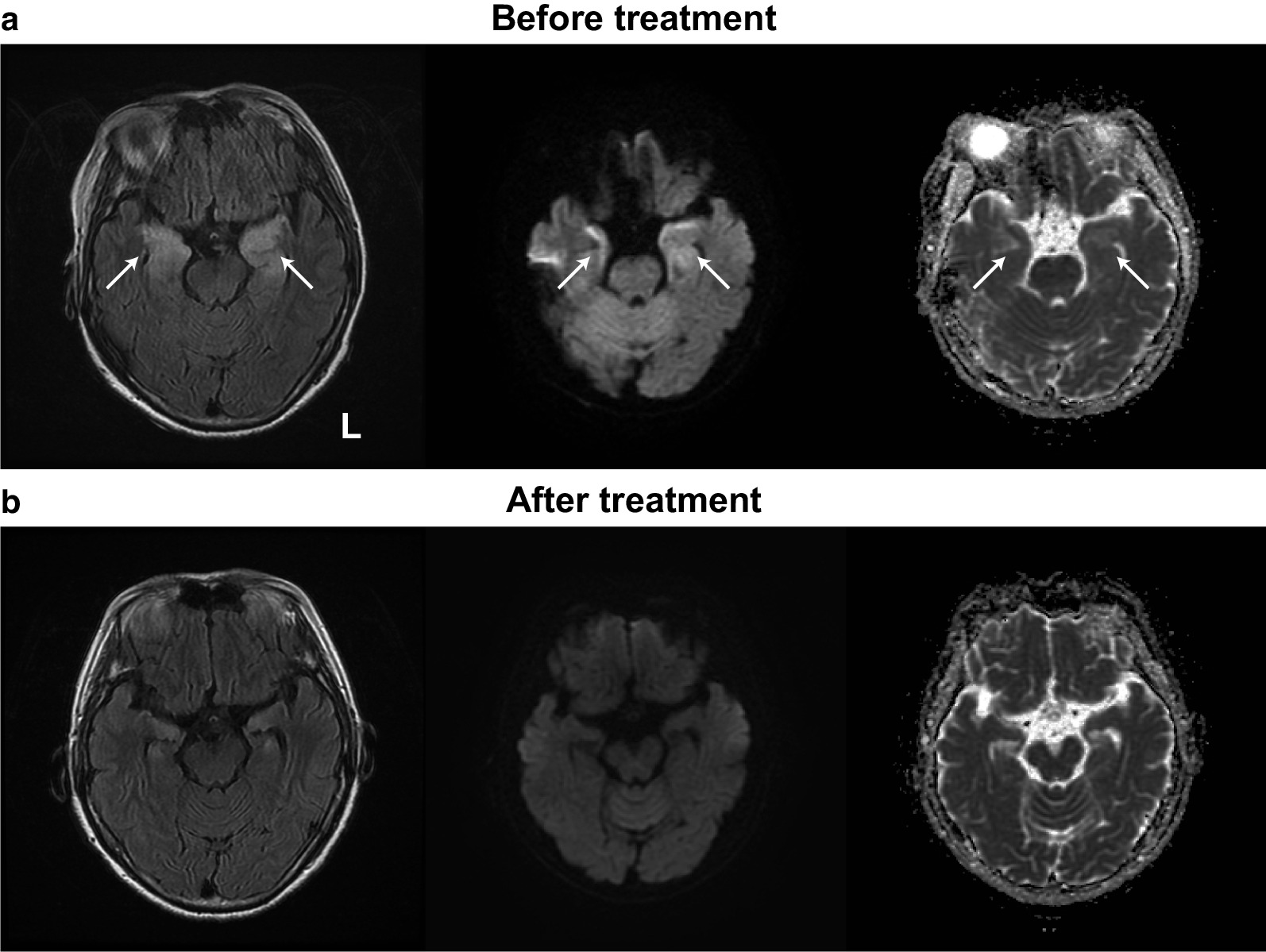
Brain MRI findings. **a**: The initial MRI (1 week after admission). Before treatment, brain MRI showed abnormal hyperintensity areas in the bilateral medial temporal lobes (arrows) on FLAIR sequence (left). Diffusion-weighted imaging (middle) and apparent diffusion coefficient map (right) also show abnormal hyperintensity in these regions. **b**: Follow-up MRI (2 months after admission). After treatment, the abnormal hyperintensity in the initial MRI was resolved

**Fig. 3:**
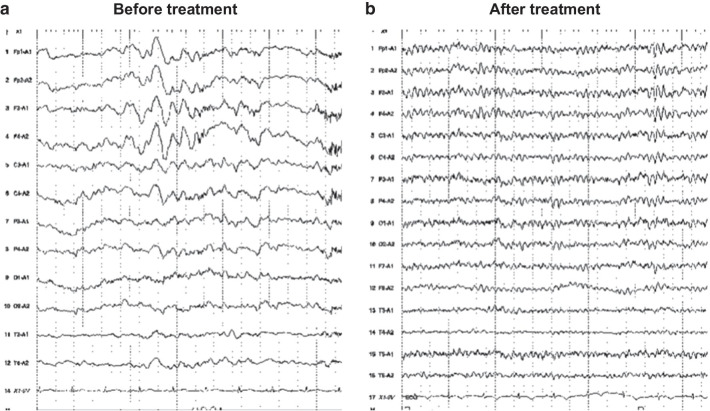
EEG findings. **a**: The initial EEG (1 week after admission). Before treatment, the EEG showed slow-wave abnormalities (2–6 Hz polymorphic delta and theta activity) without epileptic activity. **b**: Follow-up EEG (1 month after admission). After treatment, these abnormalities were resolved

**Fig. 4:**
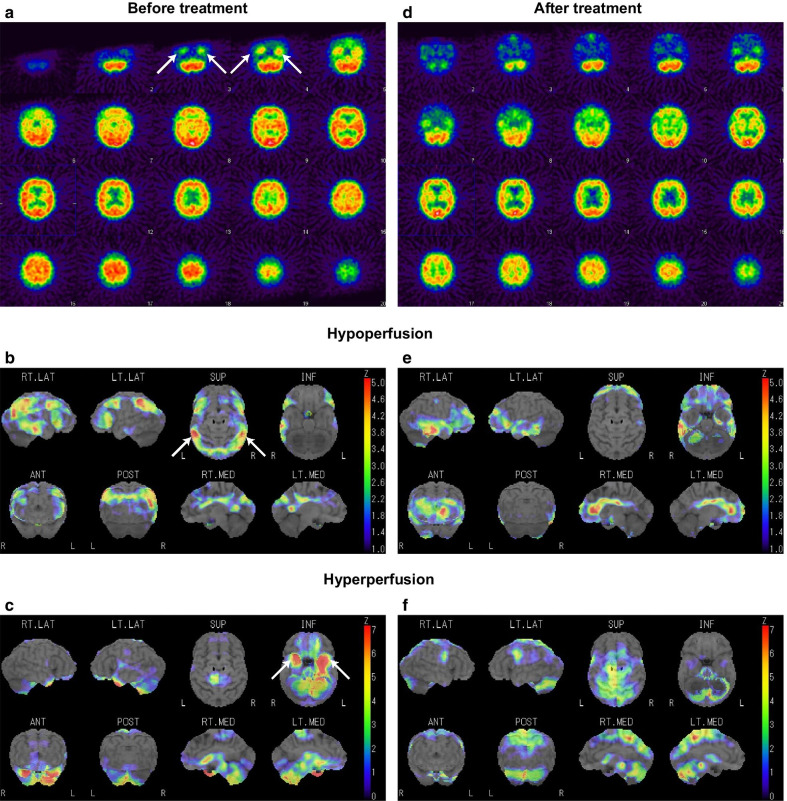
SPECT findings. **a-c**: The initial IMP-SPECT (1 week after admission). Before treatment, the abnormal bilateral hyperperfusion of the medial temporal lobes (arrows in **a** and **c**), as well as the bilateral hypoperfusion in the parietal regions (arrows in **b**), were observed. **d-f**: Follow-up IMP-SPECT (2 months after admission). After treatment, these abnormalities were resolved

Three days after the improvement of the disturbed consciousness and Takotsubo syndrome symptoms (i.e., 1 week after admission), her mental status was apparently normal. However, her brother noticed something wrong with her behavior during his visit to the hospital. Subsequently, we consulted the neurology department 1 week after admission. Her brother revealed a history of abnormal behavior by the patient (such as mistaken entry into the wrong apartment in her building or into another person’s car) a few days prior to the onset of disturbed consciousness, suggesting disorientation of place. Neurological examination showed immediate memory loss with better long-term memory retention. She scored 1 on the Rivermead behavioral memory test, which was suggestive of severe memory loss. On the revised Hasegawa’s dementia scale (HDS-R: the general cognitive test frequently used in Japan; cutoff: 20/30), she scored 9/30 with severe short-term memory loss and preserved working memory. There were no motor or sensory symptoms. Brain magnetic resonance imaging (MRI) showed an increased signal in the medial aspect of the temporal lobes, which was most clearly observed on the fluid-attenuated inversion recovery (FLAIR) sequence (Fig. 2). An electroencephalogram (EEG) showed slow-wave abnormalities (2–6 Hz polymorphic delta and theta activity) with no epileptic activity (Fig. 3). 123-iodoamphetamine single photon emission computed tomography (IMP-SPECT) showed abnormal hyperperfusion in bilateral temporal regions (Fig. 4a). A three-dimensional stereotactic surface projections (3D-SSP) analysis [5] of the IMP-SPECT data, in which the regional cerebral blood flow of the patient was compared with that of the normal control database using the *z* -test, clearly showed hypoperfusion in bilateral parietal lobes (Fig. 4b) and abnormal hyperperfusion in bilateral medial temporal lobes (Fig. 4c). She had high levels of antithyroid peroxidase antibody (192.0 IU/mL; normal: < 16.0 IU/mL) with almost normal levels of thyroid-stimulating hormone (0.485 μIU/mL; normal range: 0.5000–5.000 μIU/mL), free triiodothyronine (1.19 ng/mL; normal range: 2.30–4.00 ng/mL), and free thyroxine (1.6 pg/mL; normal range: 0.90–1.70 pg/mL). A cerebrospinal fluid (CSF) analysis revealed mild lymphocytic pleocytosis (7 cells/mm^3^; normal: < 5 cells/mm^3^) and elevated protein level (50 mg/dL; normal range: 10–40 mg/dL) with no oligoclonal bands. IgG index was normal (0.47: normal < 0.60). A polymerase chain reaction for herpes simplex virus deoxyribonucleic acid (DNA) was negative for the cerebrospinal fluid. The results of the following tests of serum sample were all negative: anti-nuclear antibody, anti-ds-DNA, SS-A, SS-B, anti N-methyl-d-aspartate (NMDA) receptor antibody, voltage-gated potassium channel-complex antibodies (leucine-rich glioma-inactivated 1 antibody, contactin associated protein-2 antibody), and paraneoplastic antibodies, including anti-Amphiphysin, anti-CV2, anti-Ma2/Ta, anti-Ri, anti-Yo, anti-Hu, anti-revoverin, anti-SOX1, anti-titin, anti-zic4, anti-GAD65, and anti-Tr antibodies. We diagnosed the patient with autoimmune limbic encephalitis and administered intravenous methylprednisolone (1 g/day) for 5 days.

Subsequently, there was gradual alleviation of memory loss (HDS-R: 21/30). Follow-up echocardiogram performed 20 days after admission showed normalization of the left ventricular EF (69%) and resolution of regional wall-motion abnormalities. Moreover, there was a resolution of the abnormal findings of MRI (2 months after admission, Fig. 2b), EEG (1 months after admission, Fig. 3b), and IMP-SPECT (2 months after admission, Fig. 4d**-**f). She was discharged 3 months after admission. No recurrence of cardiac or neurological symptoms has occurred in 12 months. Based on these clinical features, we finally diagnosed her with midventricular Takotsubo syndrome associated with autoimmune limbic encephalitis.

## Discussion and conclusions

A diagnosis of definite autoimmune limbic encephalitis requires fulfillment of all the following four criteria: (1) subacute onset (rapid progression over less than 3 months) of memory deficit, seizures, or psychiatric symptoms, suggesting the involvement of the limbic system; (2) bilateral brain abnormalities on T2-weighted FLAIR MRI highly restricted to the medial temporal lobes; (3) CSF pleocytosis (white blood cell count: > 5 cells per mm^3^) and/or EEG with epileptic or slow-wave activity involving the temporal lobes; and (4) reasonable exclusion of alternative causes, such as acute disseminated encephalomyelitis, anti-NMDA receptor encephalitis, Bickerstaff’s brainstem encephalitis, herpes simplex virus encephalitis, paraneoplastic syndrome, and Hashimoto’s encephalopathy [6]. Our patient met all four criteria. Regarding the SPECT data, a previous study has reported that patients with limbic encephalitis show hyperperfusion on SPECT, which correspond to hyperintense lesions on MRI [7]. Our patient also showed hyperperfusion in the bilateral medial temporal lobes on SPECT (Fig. 4a), which corresponded to the hyperintense lesions on MRI (Fig. 2a). These findings were highly suggestive of autoimmune limbic encephalitis for our case.

The clinical course and diagnostic work-up as well as the recovery of EF in the present case are more suggestive of midventricular Takotsubo syndrome, although cardiac MRI was not performed; therefore, myocarditis cannot be fully excluded. Among the central nervous system diseases, the most well-known causes of Takotsubo syndrome are epilepsy, stroke, infectious or immunological encephalitis/meningitis, migraine, and traumatic brain injury [3]. There is one case report on Takotsubo syndrome triggered by possible limbic encephalopathy with abnormal intensity in the bilateral medial temporal lobes [8]. This case showed no improvement of memory loss or confusion after treatment with intravenous immunoglobulins and high-dose steroids; moreover, a repeat lymph node biopsy demonstrated high-grade B-cell lymphoma, which suggested that the diagnosis of autoimmune limbic encephalitis was unlikely. In the present case, it is difficult to determine whether the disturbed consciousness presented with at the first visit was due to hypoxia or encephalitis because no extensive neurological workup (such as brain MRI or CSF examination) was performed at that time. Nevertheless, the history of disorientation of place prior to the onset of disturbed consciousness suggested that Takotsubo syndrome may have been triggered by autoimmune limbic encephalitis. Based on the present and past findings, this is the first reported case of Takotsubo syndrome possibly triggered by autoimmune limbic encephalitis.

Exaggerated sympathetic stimulation is hypothesized as the underlying cause of Takotsubo syndrome [9]. The limbic network (including the insula, amygdala, cingulate cortex, and hippocampus) is believed to contribute to the regulation of the autonomic nervous system [10]. A recent functional MRI study has demonstrated hypoconnectivity of the central brain regions associated with autonomic functions, including the limbic system, in patients with Takotsubo syndrome [11]. Considering these findings, it is presumed that dysfunction of the limbic system due to autoimmune limbic encephalitis is associated with exaggerated sympathetic stimulation, which results in Takotsubo syndrome. One possible mechanism by which exaggerated sympathetic stimulation induces Takotsubo syndrome could be attributed to its association with the spillover of stress-related neuropeptides [12]. There is a complex neocortical and limbic integration in response to stress through the activation of brainstem noradrenergic neurons and stress-related neuropeptides (i.e., neuropeptide Y (NPY) produced by the arcuate nucleus in the hypothalamus). Norepinephrine and NPY are stored in the presynaptic terminals of the postganglionic sympathetic system. Acute spillage of norepinephrine and NPY at the myocardial level, through direct catecholamine toxicity and/or microvascular dysfunction, may explain the prevailing theory of neurogenic-mediated mechanism of myocardial stunning. Patients with Takotsubo syndrome secondary to central nervous system diseases had the worst prognosis, whereas those with Takotsubo syndrome related to emotional stress showed the most favorable outcome [13–15]. Thus, it is clinically important to clarify whether diseases of the limbic system indeed cause Takotsubo syndrome. Further studies are required to elucidate the pathophysiology of Takotsubo syndrome associated with the brain–heart axis.

## Supplementary information

**Additional file 1**. Left ventriculography. Note akinesia in the middle section of the left ventricle and hyperkinesia in the apical and basal sections of the left ventricle.

## Data Availability

The datasets used and/or analyzed during the current study are available from the corresponding author on reasonable request.

## Abbreviations

BNP: Brain natriuretic peptide
DNA: Deoxyribonucleic acid
ECG: Electrocardiogram
EEG: Electroencephalogram
EF: Ejection fraction
FLAIR: Fluid-attenuated inversion recovery
HDS-R: Revised Hasegawa’s dementia scale
IMP-SPECT: 123-iodoamphetamine single-photon emission computed tomography
MRI: Magnetic resonance imaging
NMDA: N-methyl-d-aspartate
NPY: Neuropeptide Y
NPPV: Noninvasive positive pressure ventilation
3D-SSP: Three-dimensional stereotactic surface projections
